# Supplementary material for: Inhibition of 7-dehydrocholesterol reductase prevents hepatic ferroptosis under an active state of sterol synthesis
Source: Nat Commun. 2024 Mar 12;15:2195. doi: 10.1038/s41467-024-46386-6 (PMC10933264; doi:10.1038/s41467-024-46386-6)
Supplement: Supplementary file 1 — Supplementary Information [file 41467_2024_46386_MOESM1_ESM.pdf]

Supplementary information

Inhibition of 7-dehydrocholesterol reductase prevents hepatic ferroptosis under an active state of sterol synthesis

Naoya Yamada[1,2,3]*; Tadayoshi Karasawa[1]*; Junya Ito[4]; Daisuke Yamamuro[5];
Kazushi Morimoto[6]; Toshitaka Nakamura[3]; Takanori Komada[1]; Chintogtokh Baatarjav[1];
Yuma Saimoto[6]; Yuka Jinnouchi[6]; Kazuhisa Watanabe[7];
Kouichi Miura[8]; Naoya Yahagi[5]; Kiyotaka Nakagawa[4]; Takayoshi Matsumura[1,7];
Ken-ichi Yamada[6]; Shun Ishibashi[5]; Naohiro Sata[2]; Marcus Conrad[3]; Masafumi Takahashi[1]

[1]Division of Inflammation Research, Center for Molecular Medicine,
Jichi Medical University, Shimotsuke, Tochigi, Japan.
[2]Division of Gastroenterological, General and Transplant Surgery, Department of Surgery,
Jichi Medical University, Shimotsuke, Tochigi, Japan.
[3]Institute of Metabolism and Cell Death, Molecular Target and Therapeutics Center, Helmholtz Munich, Neuherberg, Bavaria, Germany
[4]Laboratory of Food Function Analysis, Graduate School of Agricultural Science,
Tohoku University, Sendai, Miyagi, Japan.
[5]Division of Endocrinology and Metabolism, Department of Medicine,
Jichi Medical University, Shimotsuke, Tochigi, Japan.
[6]Department of Molecular Pathobiology, Faculty of Pharmaceutical Sciences,
Kyushu University, Fukuoka, Fukuoka, Japan.
[7]Division of Human Genetics, Center for Molecular Medicine,
Jichi Medical University, Shimotsuke, Tochigi, Japan.
[8]Division of Gastroenterology, Department of Medicine,
Jichi Medical University, Shimotsuke, Tochigi, Japan.

* These authors contributed equally.
Correspondence: Naoya Yamada, MD, PhD, Tadayoshi Karasawa, PhD,
or Masafumi Takahashi, MD, PhD, FACP, FAHA

Division of Inflammation Research, Center for Molecular Medicine, Jichi Medical University
3311-1 Yakushiji, Shimotsuke, Tochigi 329-0498, Japan, E-mail: naoya.yamada@helmholtz-munich.de, tdys.karasawa@jichi.ac.jp, or masafumi2@jichi.ac.jp

Supplementary Fig.1

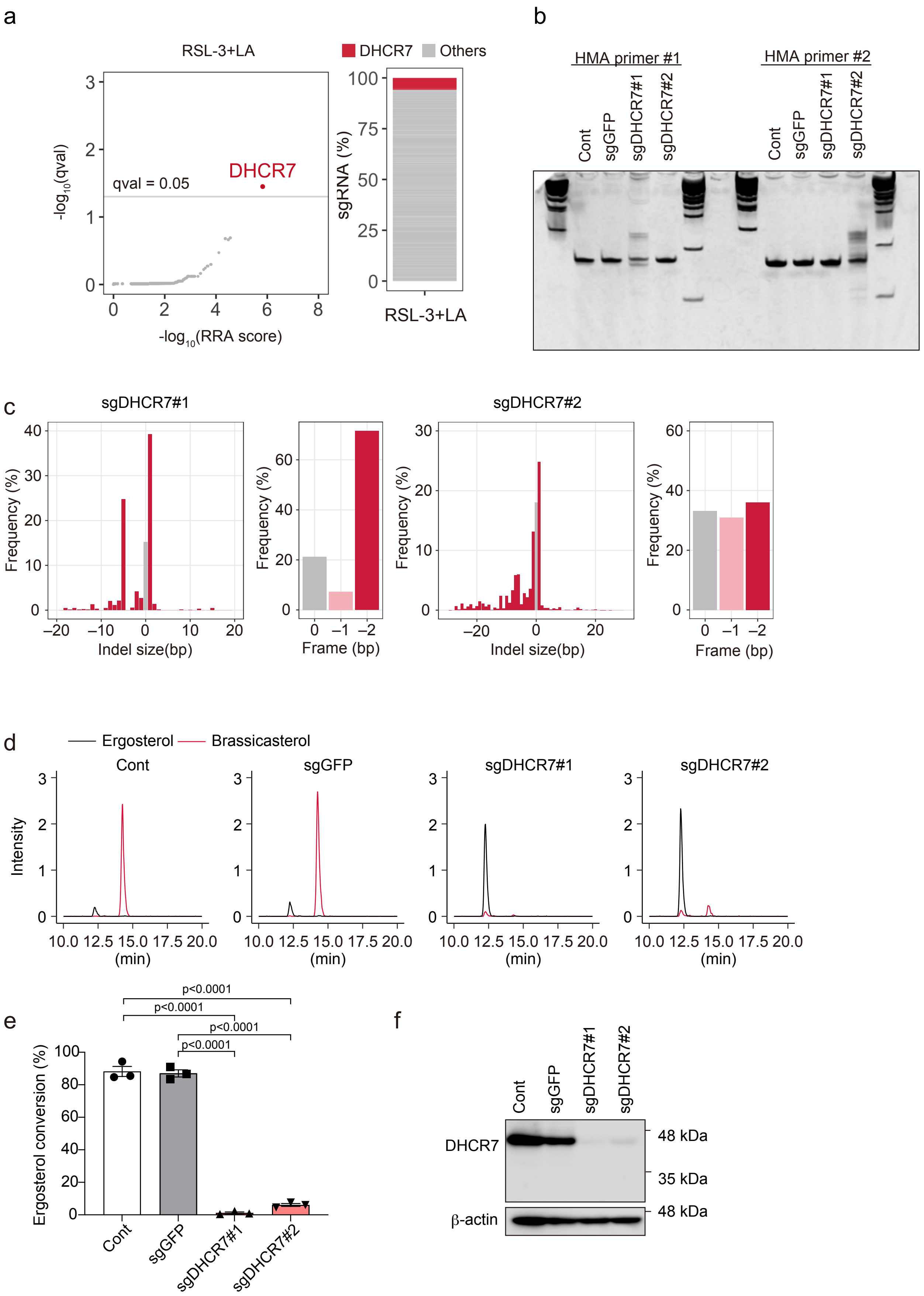

Supplementary Fig. 1. Genetic deletion of DHCR7 suppresses ferroptosis in Huh-7 cells

(a) Huh-7 cells were treated with RSL-3 (0.03 μM) with LA (50 mM) for 24 h. The ferroptosis-resistant cells were selected 4 times, and the genomic DNA was purified and analyzed by NGS. (b and c) *DHCR7*-ablated Huh-7 cells were generated using the CRISPR/Cas9 genome editing system. (b) Indel mutations of each sgRNA analyzed by the HMA method. (c) The frequency of indel was analyzed by amplicon sequencing and subsequent CRISPResso2 analysis. (d and e) Enzymatic activities of DHCR7 was assessed by evaluating conversion of ergosterol to brassicasterol. DHCR7-ablated Huh-7 cells were treated with 2 μg/mL ergosterol for 24 h. (d) Cellular ergosterol levels (*Er*) and brassicasterol levels (*Br*) were assessed by LC-MS/MS. (e) The efficiency of ergosterol conversion was calculated as *Br* / *Br* + *Er*. (f) Expression of DHCR7 was assessed by Western blot. (d and f) Data are representative of (d) three or (f) two experiments. (e) Data are means ± SEM of three independent experiments and expressed as dot plots. Statistical significance was calculated using one-way ANOVA with Tukey's post hoc test.

Supplementary Fig.2

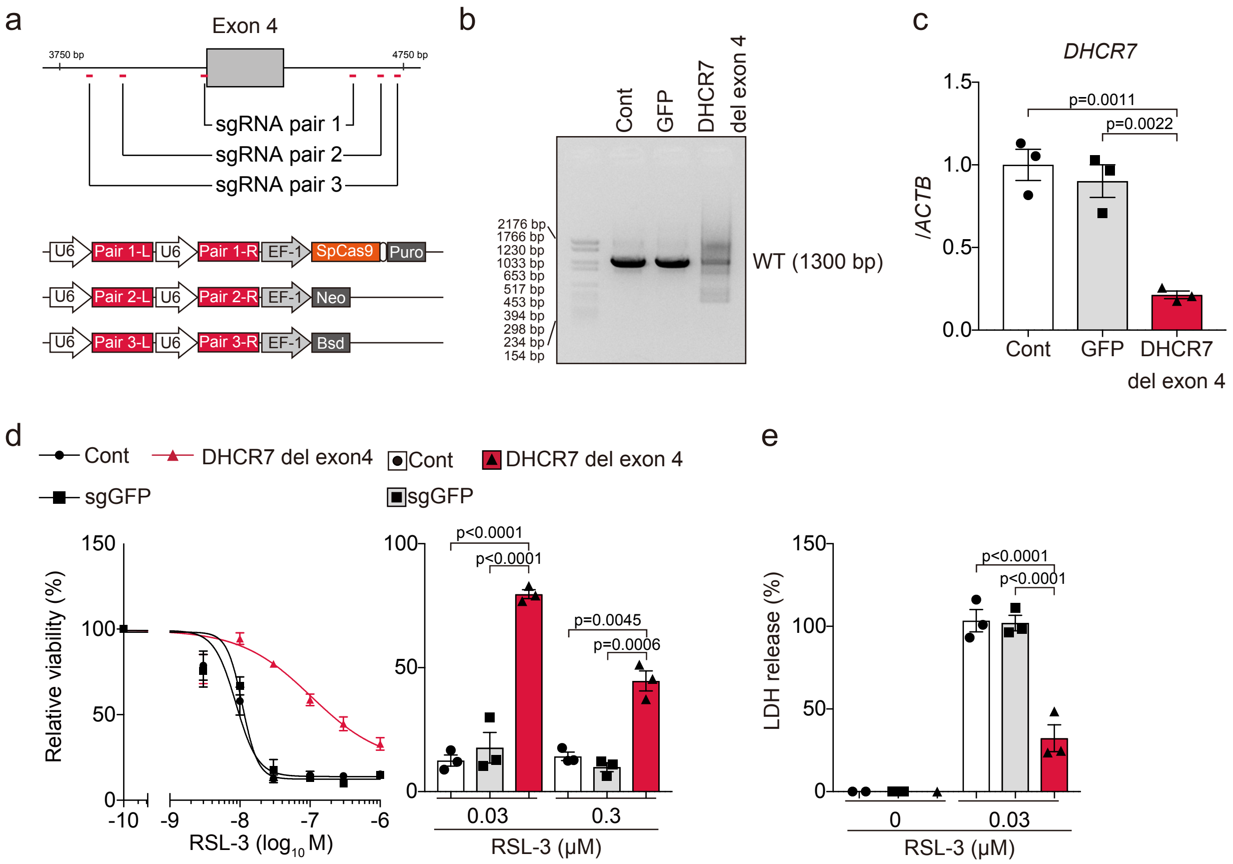

Supplementary Fig. 2. Deletion of exon 4 in DHCR7 gene suppresses ferroptosis in Huh-7 cells

(a–e) Huh-7 cells lacking the exon 4 of *DHCR7*. (a) Schematic diagram of the gRNA targeting site on the human *DHCR7* gene. The exon 4 of *DHCR7* were deleted by multiple transductions of sgRNA pair targeting *DHCR7*. (b) Disruption of the *DHCR7* gene was confirmed by amplification of the targeted region. (c) The mRNA levels of *DHCR7* were assessed by real-time RT-PCR analysis. (d and e) Control (GFP) and *DHCR7* KO Huh-7 cells were treated with or without RSL-3 for 24 h. Cytotoxicity and cell death were assessed by an (d) MTT and (e) LDH release assays. (c–e) Data are means of three independent experiments and expressed as dot plots and means ± SEM. Statistical significance was calculated using one-way or two-way ANOVA with Tukey's post hoc test.

Supplementary Fig. 3

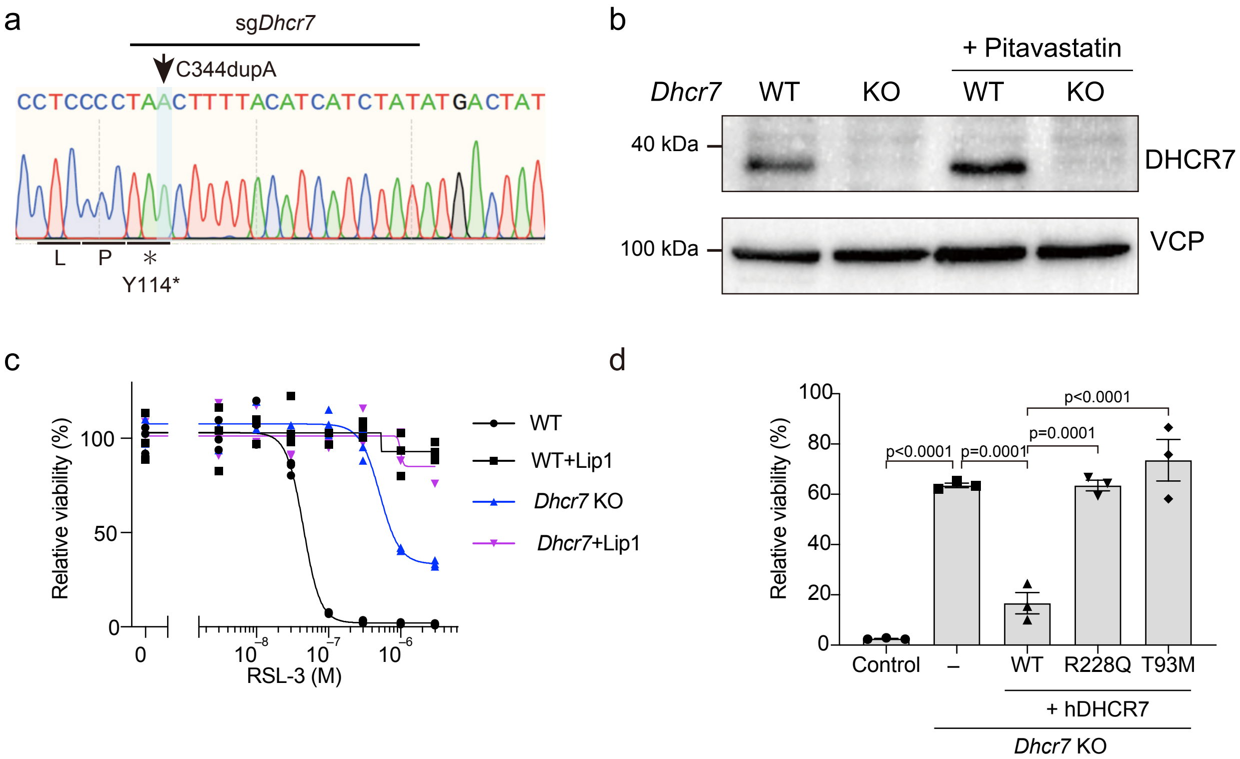

Supplementary Fig. 3. Genetic deletion of murine Dhcr7 suppresses ferroptosis in Pfa1 cells

(a) A mutation in *Dhcr7* KO Pfa1 cells was confirmed by Sanger sequencing. (b) Expression of DHCR7 was assessed by Western blot. (c) Control and *Dhcr7*-KO Pfa1 cells were treated with RSL-3 in the presence or absence of Lip-1 (0.5 μM) for 24 h. Cell viability was assessed by the resazurin assay. (d) Control and *Dhcr7*-KO Pfa1 cells were transduced with WT or mutated (T93M, R228Q) hDHCR7 and treated with RSL-3 (100 nM) for 24 h. Cell viability was assessed by the resazurin assay. Data are expressed as dot plots and means ± SEM. Statistical significance was calculated using one-way ANOVA with Tukey's post hoc test.

Supplementary Fig.4

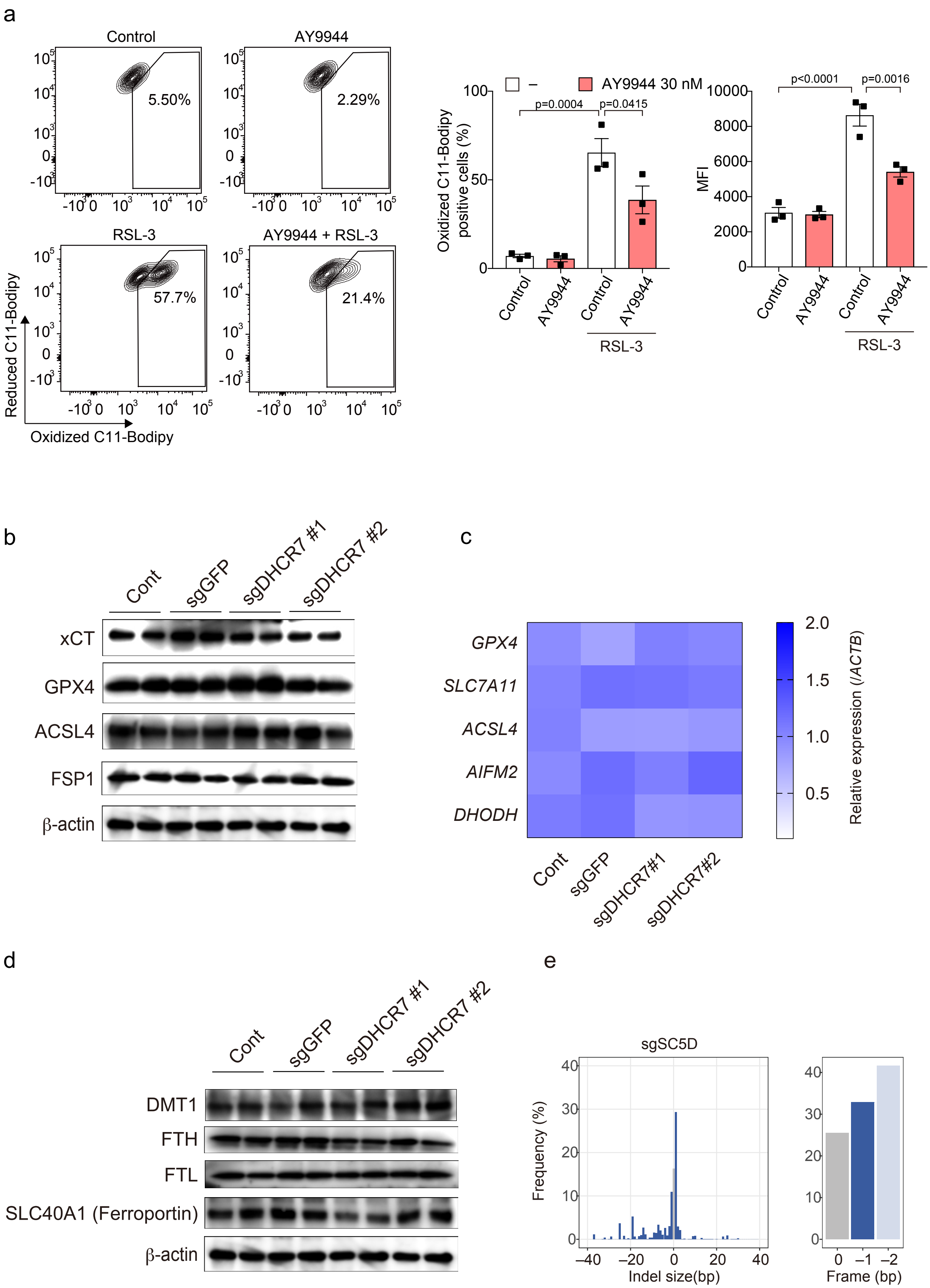

Supplementary Fig. 4. Evaluation of lipid peroxidation and ferroptosis-regulatory molecules in DHCR7-ablated Huh-7 cells

(a) Huh-7 cells were treated with RSL-3 (0.1 μM) for 6 h with or without AY9944 (30 nM) 1 h prior to RSL-3 administration. The fluorescence intensity of C11-BODIPY$^{581/591}$ was analyzed by flow cytometry. The number of oxidized C11-BODIPY$^{581/591}$-positive cells and mean fluorescence intensity of oxidized C11-BODIPY$^{581/591}$ were analyzed. (b–d) Proteins or RNA was isolated from control (sgGFP) and *DHCR7*-ablated (sgDHCR7) Huh-7 cells. (b) Proteins involved in ferroptosis regulation were assessed by Western blot. (c) Expression of genes involved in ferroptosis regulation (*GPX4, SLC7A11, ACSL4, AIFM2, and DHODH*) were assessed by real-time RT-PCR analysis. (d) Proteins involved in iron metabolism were assessed by Western blot. (e) *DHCR7*- and *SC5D*-ablated Huh-7 cells were generated using the CRISPR/Cas9 genome editing system. The frequency of indel was analyzed by amplicon sequencing and subsequent CRISPResso2 analysis. (a) Data are means of three independent experiments and expressed as dot plots and means ± SEM. Statistical significance was calculated using two-way ANOVA with Tukey's post hoc test.

Supplementary Fig.5

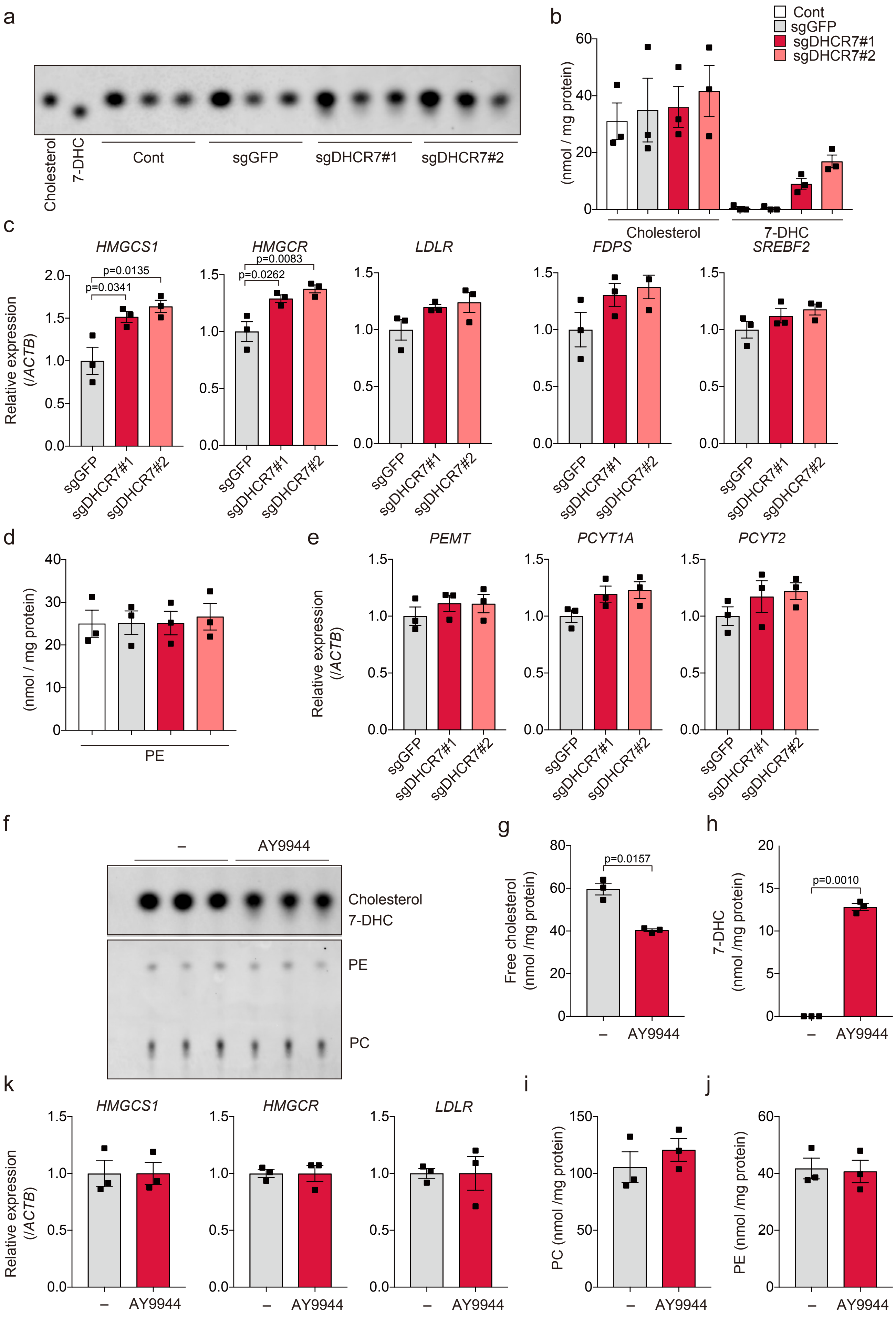

Supplementary Fig. 5. Lipid profiles in DHCR7-ablated Huh-7 cells

(a–e) Total lipids and RNA were extracted from control (sgGFP) and *DHCR7*-ablated (sgDHCR7) Huh-7 cells. (a and b) Lipids were analyzed by thin layer chromatography. (b) Spots of cholesterol and 7-DHC were semi-quantified. (c) Expression of genes involved in cholesterol metabolism (*HMGCS1, HMGCR, LDLR, FDPS,* and *SREBF2*) was assessed by real-time RT-PCR analysis. (d) Levels of phosphatidyl ethanolamine (PE) were determined by enzymatic assay. (e) Expression of genes involved in cholesterol metabolism (*PEMT, PCYT1A, and PCYT2*), were assessed by real-time RT-PCR analysis. (F–K) Huh7 cells were treated with AY9944 (100 nM) for 24 h. (f) Total lipids were extracted and analyzed by thin layer chromatography. (g) Spots of cholesterol, (h) 7-DHC, (i) phosphatidylcholine (PC), and (j) phosphatidylethanolamine were semi-quantified. (k) Expression levels of genes involved in cholesterol metabolism (*HMGCS1, HMGCR,* and *LDLR*) were assessed by real-time RT-PCR analysis. Statistical significance was calculated using one-way ANOVA with Tukey's post hoc test. (a–j) Data are collected from three independent experiments and expressed as dot plots and means ± SEM. Statistical significance was calculated using one-way ANOVA with Tukey's post hoc test or two-tailed Student's t-test.

Supplementary Fig.6

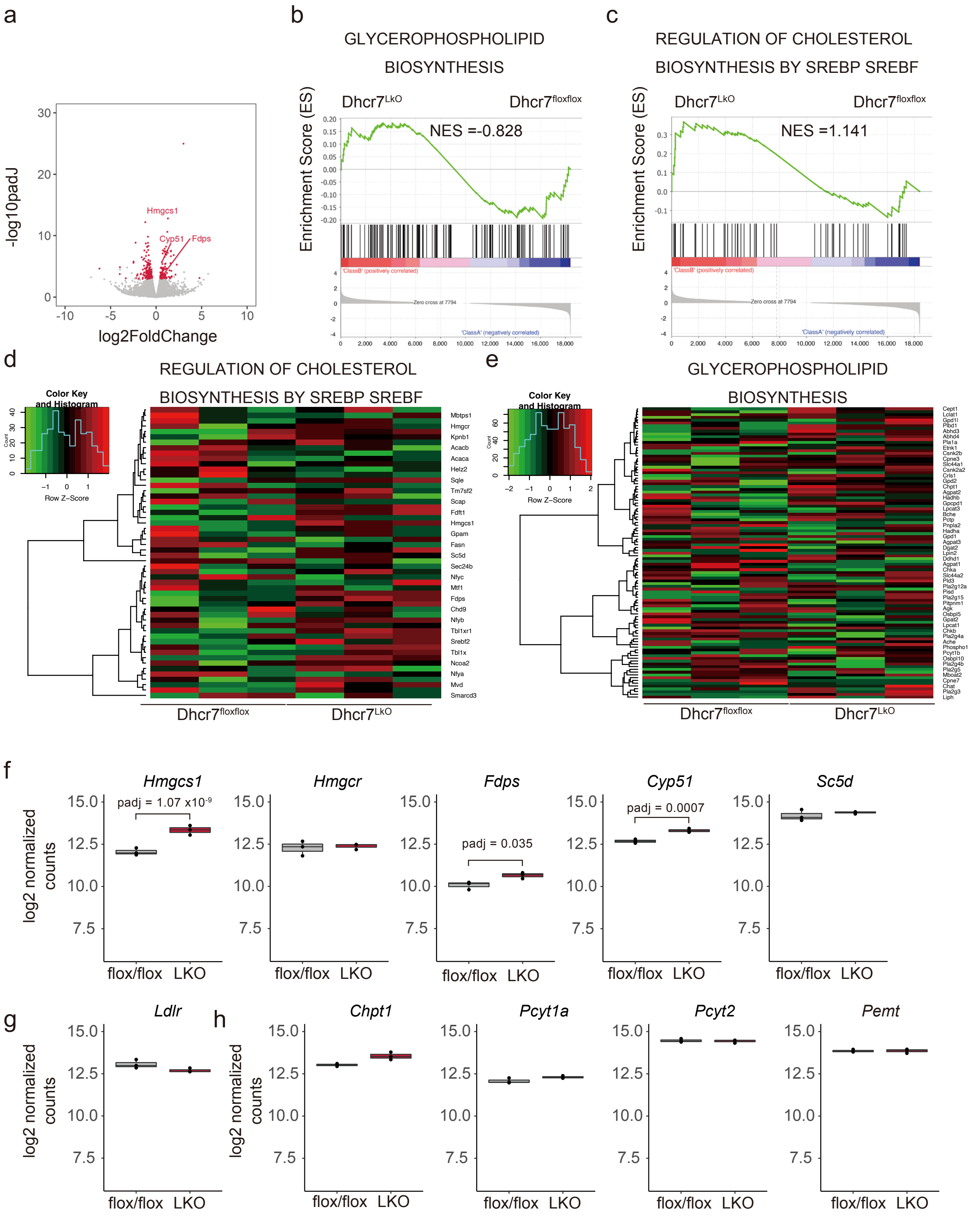

Supplementary Fig. 6. Gene expression profiles of lipid metabolism in liver- specific Dhcr7 knockout mice

(a–g) RNA-seq analysis of liver-specific *Dhcr7* knockout (*Dhcr7* LKO) mice. RNA was isolated from the liver of male Dhcr7$^{flox/flox}$ mice and Alb-Cre+ Dhcr7$^{flox/flox}$ mice (PRJNA610810). (a) Volcano plot of all genes. The red plots represent significantly upregulated or downregulated genes in *Dhcr7* LKO mice. Significantly upregulated genes listed in the Regulation of cholesterol biosynthesis are labeled. (b and c) GSEA was performed using Reactome gene sets comparing male Dhcr7$^{flox/flox}$ mice and Alb-Cre+ Dhcr7$^{flox/flox}$ mice. The enrichment plot of (b) Glycerophospholipid biosynthesis and (c) Regulation of cholesterol biosynthesis by SREBP. (d and e) Heatmap showing the expression levels of (d) Glycerophospholipid biosynthesis and (e) Regulation of cholesterol biosynthesis by SREBP. (f) Expression of genes involved in cholesterol metabolism (*Hmgcs1*, *Hmgcr, Fdps, Cyp51,* and *Sc5d*), (g) lipoprotein uptake (*Ldlr*), and (h) phospholipid metabolism (*Chpt1, Pcyt1a, Pcyt2,* and *Pemt*). Normalized expression in each group is shown. (a-g) Data from 3 mice per group were analyzed.

Supplementary Fig. 7

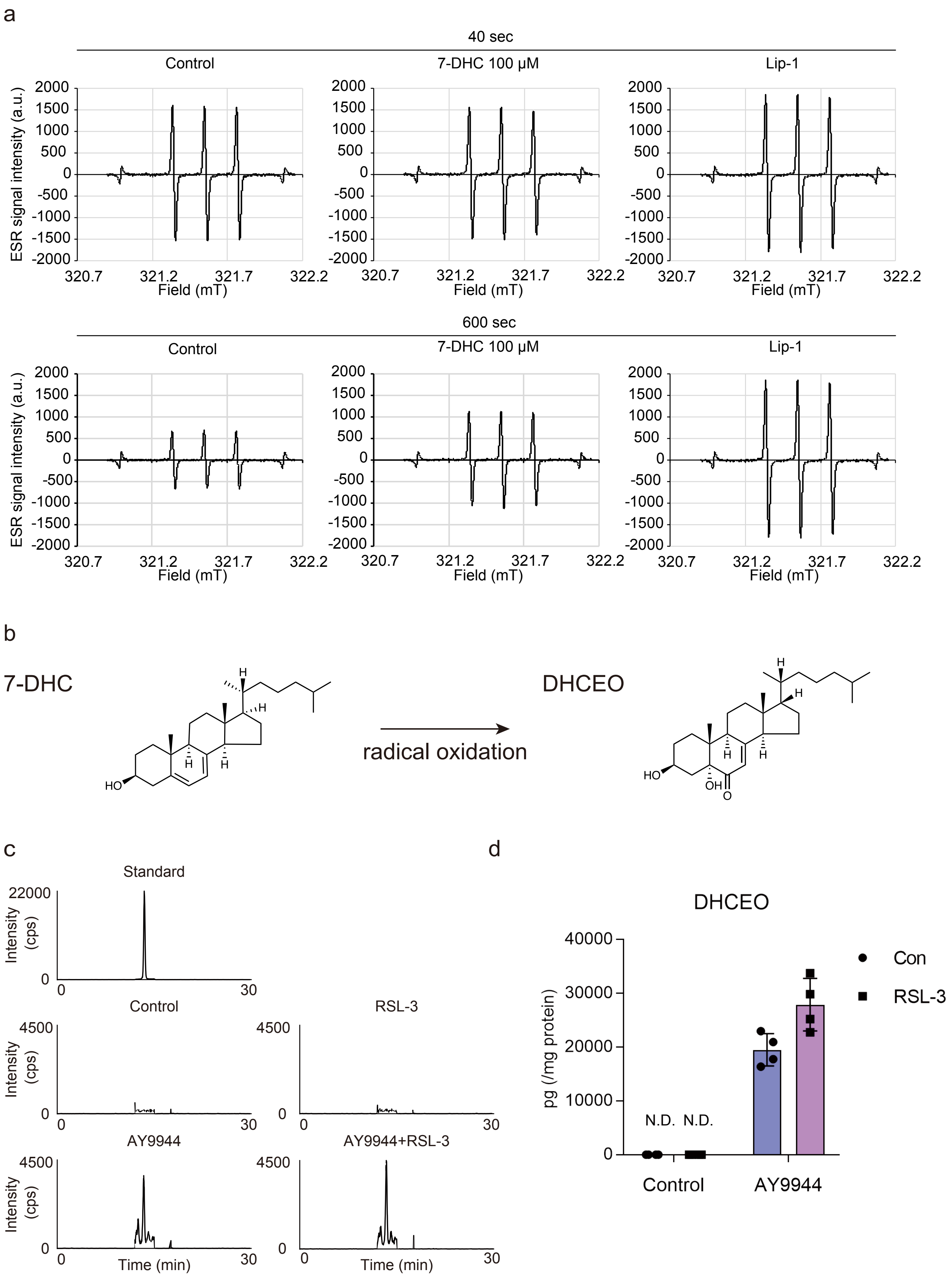

Supplementary Fig. 7. Analysis of DHCEO in AY9944-treated Huh-7 cells

(a) ESR signal intensity of TEMPOL (50 μM) after the addition of LOX (15 μg/ml) and AA (250 μM) with 7-DHC-HPβCD (100 μM), or Lip-1 (100 μM) were analyzed. The representative ESR spectra of TEMPOL in the presence of 7-DHC or Lip-1. (b) DHCEO is a major radical oxidation product of 7-DHC. (c and d) Huh-7 cells were treated with AY9944 (30 nM) for 1 h, followed by RSL-3 (0.1 μM) for 16 h. Intracellular DHCEO levels were assessed using LC-MS-MS analysis. Data are from four replicates and expressed as dot plots and means ± SEM.

Supplementary Fig.8

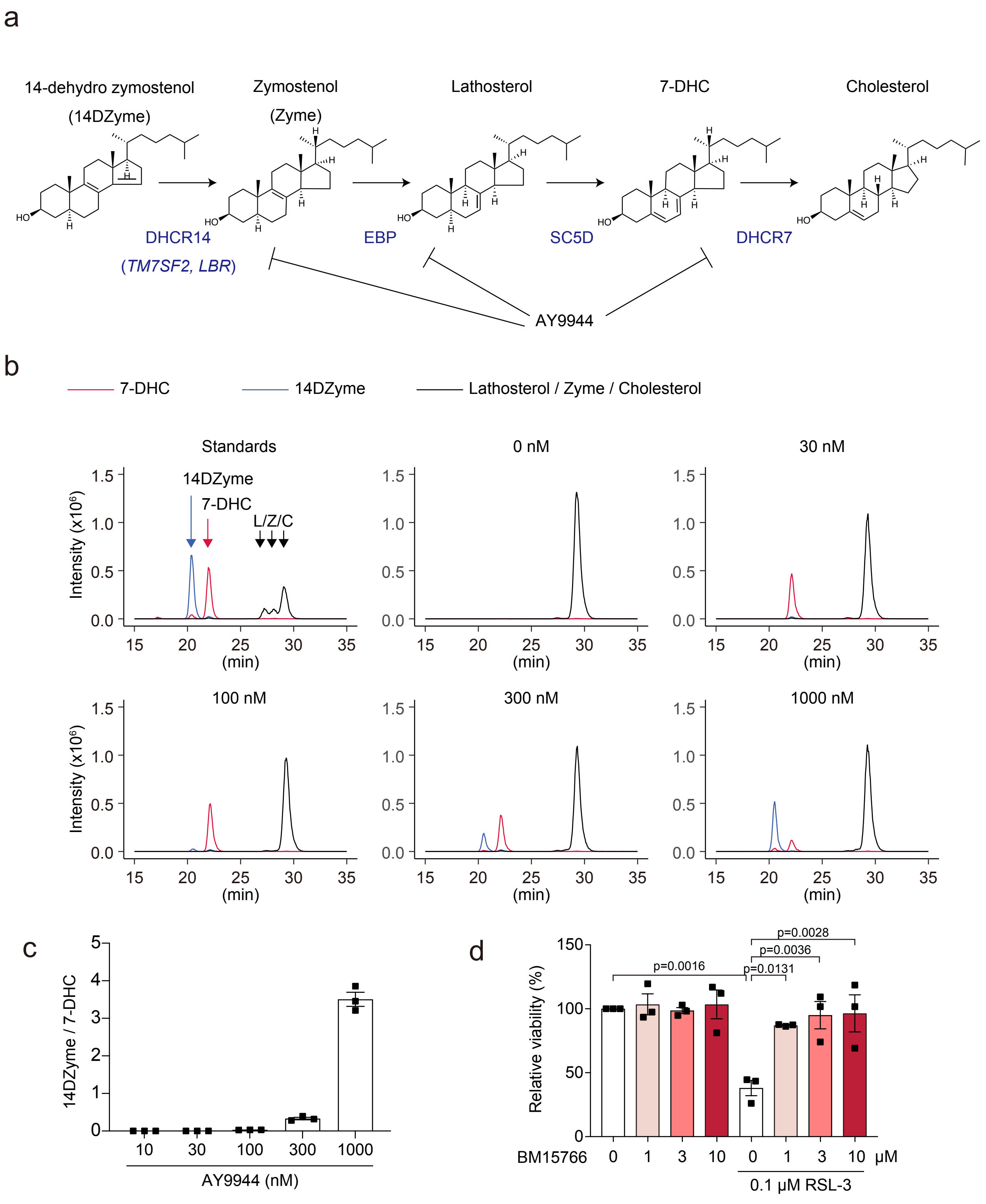

Supplementary Fig. 8. Effects of AY9944 on 14-dehydro zymostenol levels in Huh-7 cells

(a) Schema of the potential target of AY9944 in the cholesterol synthetic pathway. (b and c) Huh-7 cells were treated with AY9944 (10–1000 nM) for 24 h. The profile of sterol intermediates was analyzed by LC-MS/MS. (b) Representative chromatogram of standards (L: lathosterol, Z: zymostenol, C: cholesterol) and AY9944-tretaed cells. (c) The ratio of 14-dehydro zymostenol and 7-DHC. (d) Huh-7 cells were pretreated with BM15766 for 1 h, followed by RSL-3 treatment for 24 h. Cell viability was assessed by the MTT assay. (b-d) Data are (b) representative or (c and d) means of three independent experiments and expressed as dot plots and means ± SEM. Statistical significance was calculated using two-way ANOVA with Tukey's post hoc test.

Supplementary Fig.9

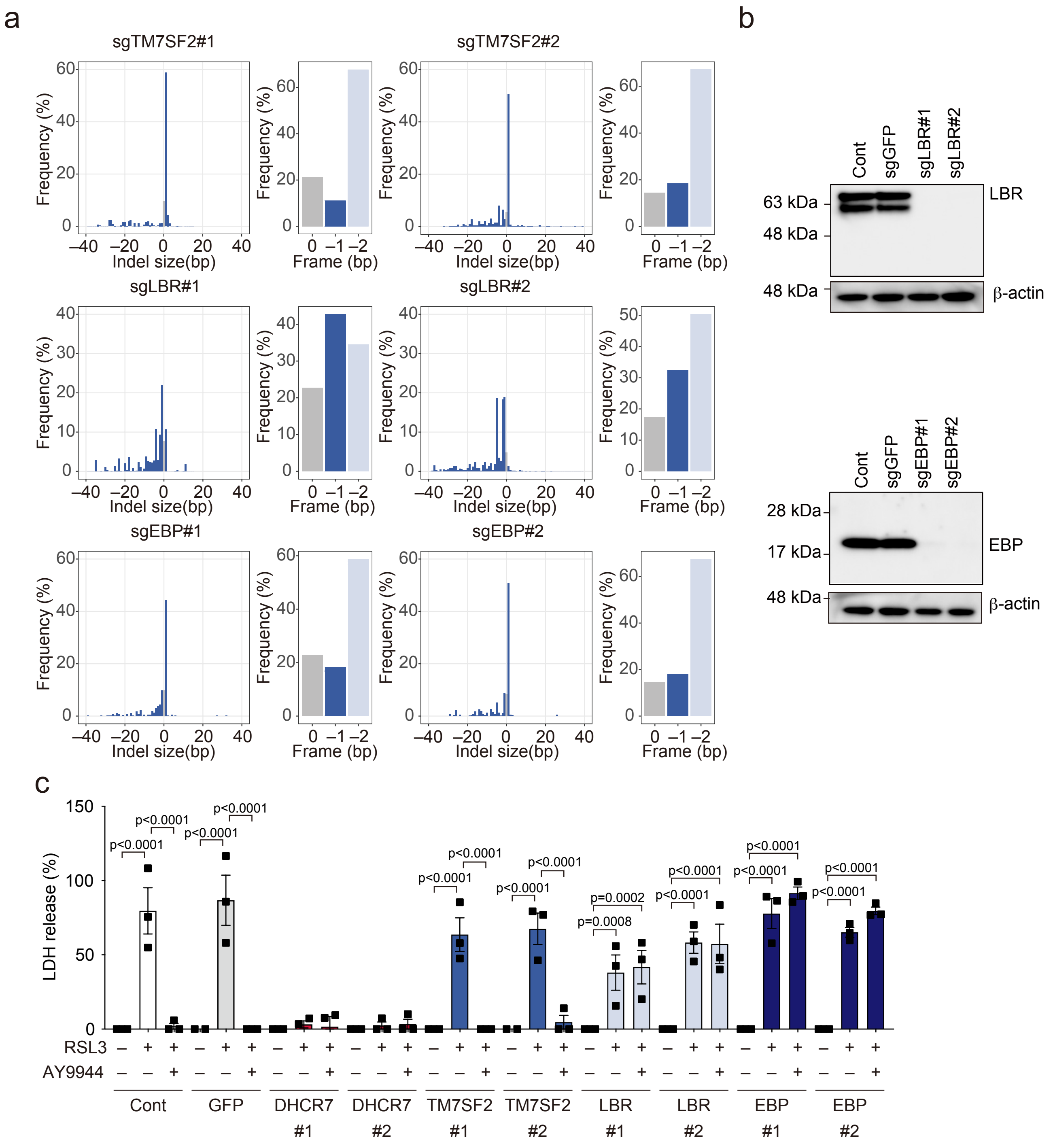

Supplementary Fig. 9. Effects of AY9944 in TMSF2-, LBR, and EBP-ablated Huh-7 cells

(a) The frequency of indel in *TM7SF2*-, *LBR*-, and *EBP*-ablated Huh-7 cells was analyzed by amplicon sequencing and subsequent CRISPResso2 analysis. (b) Expression of LBR and EBP were assessed by Western blot. (c) *DHCR7*-, *TM7SF2*-, *LBR*-, and *EBP*-ablated Huh-7 cells were treated with AY9944 (30 nM) for 1 h, followed by RSL-3 treatment for 24 h. Cell death were assessed by an LDH release assay. (c) Data are means of three independent experiments and expressed as dot plots and means ± SEM. Statistical significance was calculated using two-way ANOVA with Tukey's post hoc test.

Supplementary Fig.10

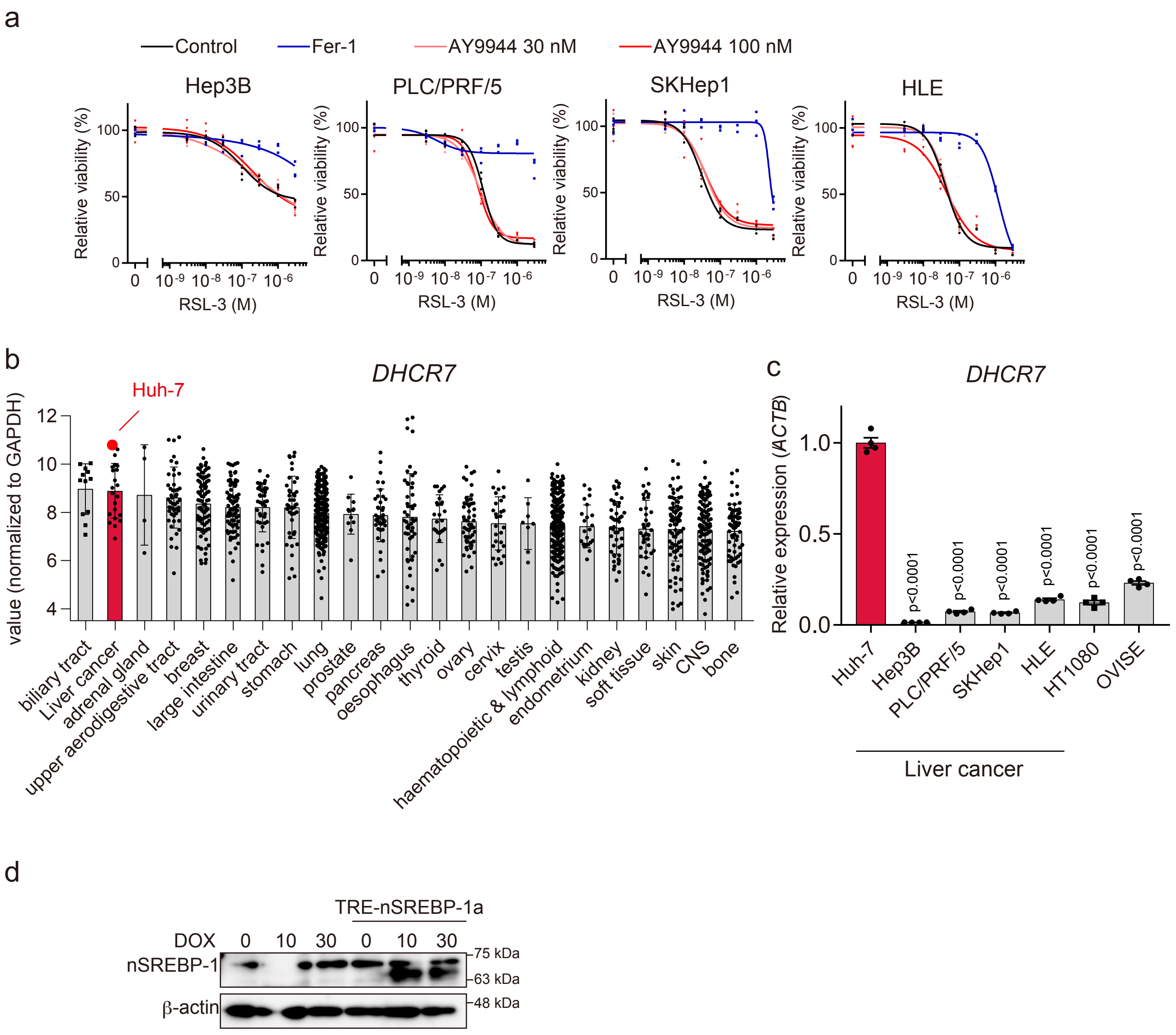

Supplementary Fig. 10. Relation between the effect of AY9944 and DHCR7 expression

(a) Cells were pretreated with Fer-1 (0.5 μM) or AY9944 (30 and 100 nM) for 1 h, and then treated with RSL-3 (0.1 μM) for 24 h. Cell viability was assessed by the MTT assay. (b) *DHCR7* expression in various cancer cells was analyzed using the CellExpress system. (c) *DHCR7* mRNA levels in ferroptosis-sensitive cancer cells were assessed. (d) TRE-nSREBP-1a–HT1080 cells were treated with DOX (10 ng/mL or 30 ng/mL) for 6 h. Expression of SREBP-1 was assessed by Western blot. (d) Data are representative of three independent experiments. (a–c) Data are expressed as dot plots and means ± SEM. Statistical significance was calculated using one-way ANOVA with Tukey's post hoc test.

Supplementary Fig. 11

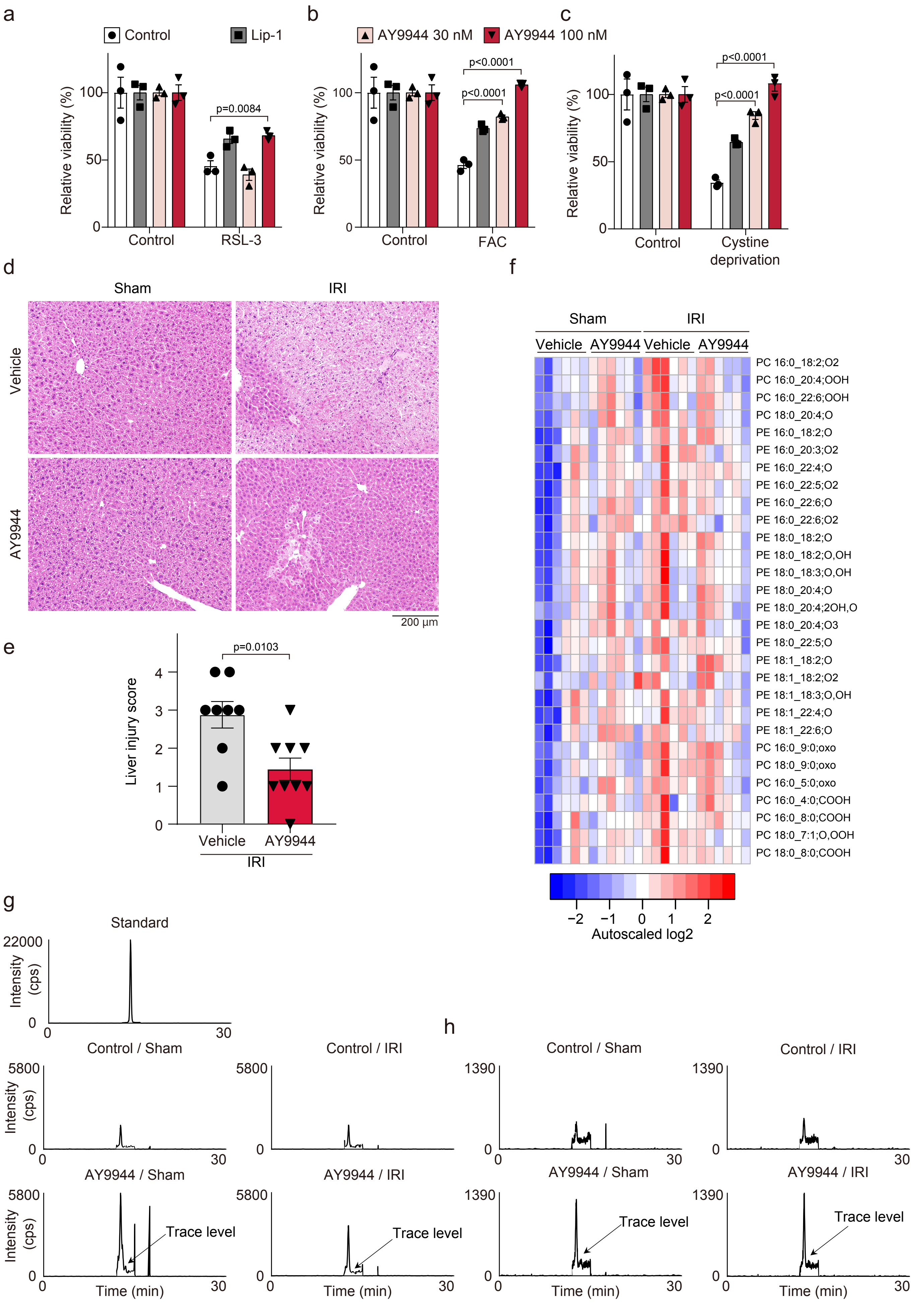

Supplementary Fig. 11. Effect of AY,9944 on ferroptosis in the liver

(a–c) Murine primary hepatocytes were isolated from male mice fed a vitamin E-deficient diet for 3 months, pretreated with Fer-1 (0.5 µM) or AY9944 (30 and 100 nM) for 1 h, and then treated with (a) RSL-3 (0.3 µM), (b) FAC (100 µM), or (c) cystine deprivation for 24 h. Cell viability was assessed by the MTT assay. (d–h) Liver samples and serum were obtained from hepatic ischemia-reperfusion or sham-operated C57BL/6J male mice treated with AY9944 (25 mg/kg) 1 h prior to open laparotomy. (d) The sectioned tissues were stained with HE and (e) the liver injury score was assessed. (f) Lipid peroxides in the liver were assessed by LC-MS/MS. (g) DHCEO levels in the liver (h) and serum were assessed using LC-MS/MS analysis. (d and e) Data are from 8 mice in vehicle group and 9 mice in AY9944 group. (f) Data are from 6 mice per group. Statistical significance was calculated using two-way ANOVA with Tukey's post hoc test or two-tailed Mann-Whitney's U test.

Supplementary Fig.12

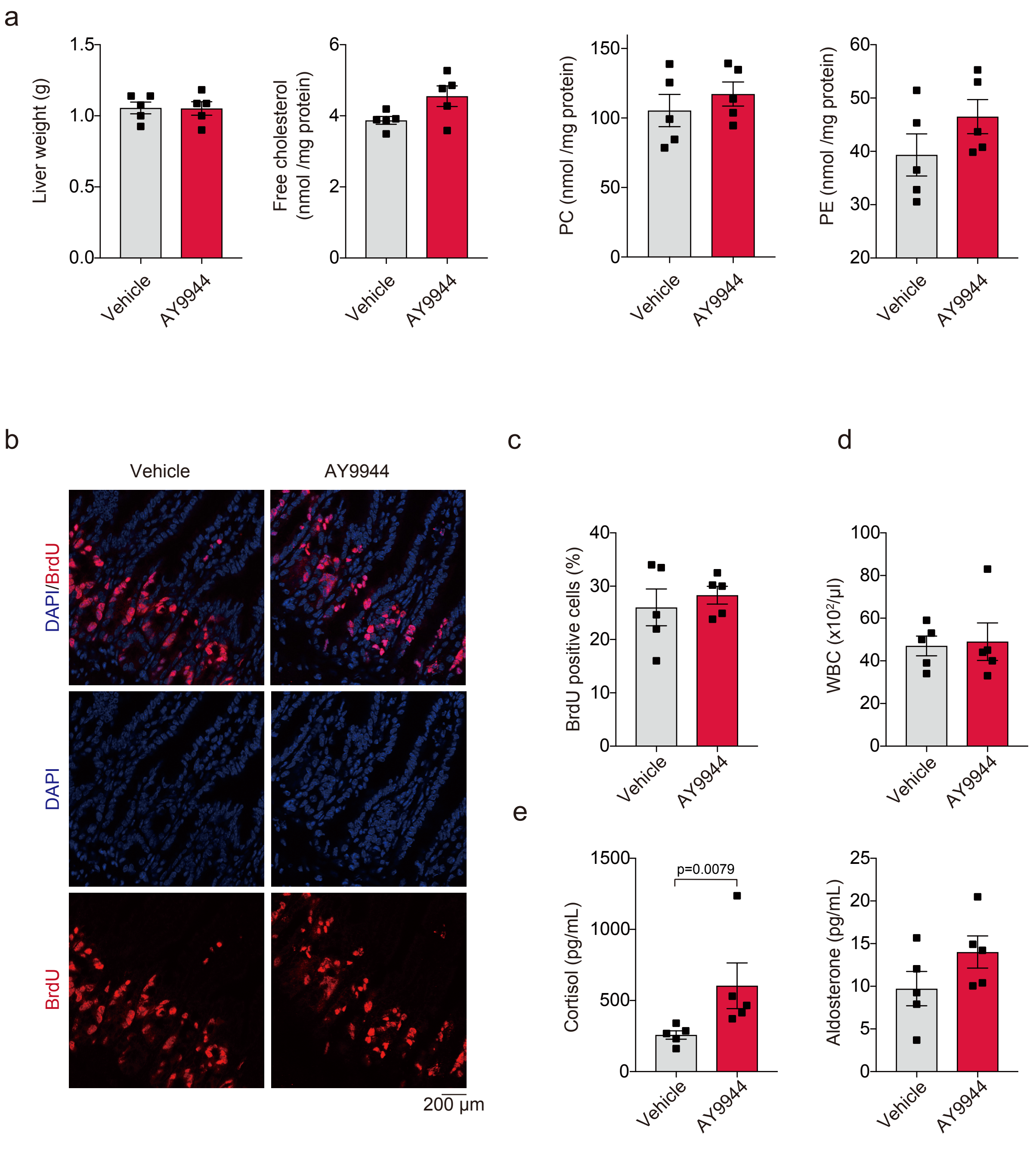

Supplementary Fig.12. Evaluation of the adverse effect in AY9944-treated mice

(a–e) C57BL/6J mice were intraperitoneally injected with either vehicle or AY9944 (25 mg/kg) and BrdU (1mg/kg). (a) Total lipids were extracted from the liver and analyzed by thin-layer chromatography. Spots of free cholesterol, phosphatidylethanolamine (PE), and phosphatidylcholine (PC) were visualized and semi-quantified. (b and c) The sections of the small intestine were stained with anti-BrdU antibody. (c) The number of BrdU-positive cells was counted. (d) The number of white blood cells in the blood. (e) The plasma cortisol and aldosterone levels were measured. Data are from 5 mice per group and expressed as dot plots and means ± SEM. Statistical significance was calculated using two-tailed Mann-Whitney's U test.

Supplementary Fig.13

a

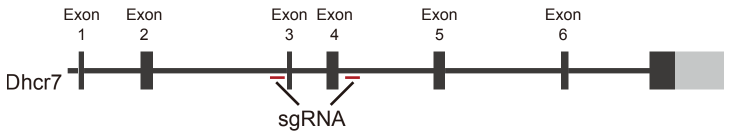

b

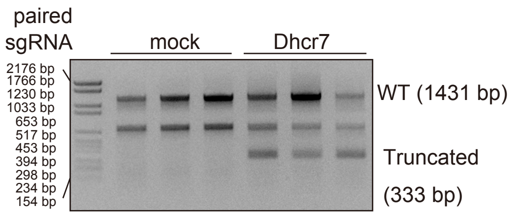

c

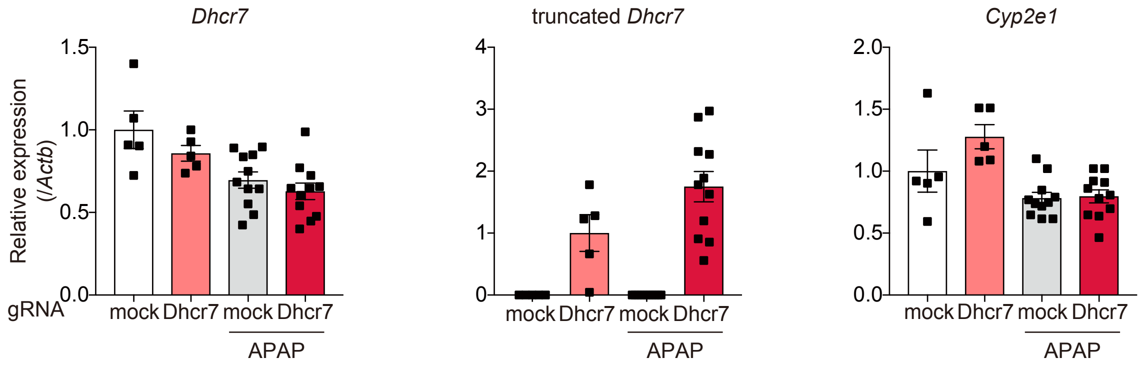

Supplementary Fig. 13. Effect of Dhcr7 gene ablation on APAP-induced liver injury

(a) Schematic diagram of the gRNA targeting site on the mouse *Dhcr7* gene. (b and c) C57BL/6J mice were injected with 10 µg of px330 carrying a pair of Dhcr7-targeting gRNA or empty gRNA scaffold (NC) using a hydrodynamics-based procedure. PBS or acetaminophen was intraperitoneally injected 7 days after gene transduction. (b) Disruption of the *Dhcr7* gene was confirmed by amplification of targeted region. (c) mRNA levels of intact *Dhcr7*, truncated *Dhcr7*, and *Cyp2e1* were assessed by real-time RT-PCR analysis. (c) Data are from 5–11 mice per group (n=5 in mock/vehicle and Dhcr7/vehicle, n=11 in mock/APAP and Dhcr7/APAP) and expressed as dot plots and means ± SEM.

Supplementary Table 1. sgRNA used for CRISPR/Cas9-mediated genome editing

	Forward	Reverse
sg*DHCR7*#1	CACCGTCAAACCACTTCCCGATCCG	AAACCGGATCGGGAAGTGGTTTGAC
sg*DHCR7*#2	CACCGTGGGCGGCTTTCCTCGTTAT	AAACATAACGAGGAAAGCCGCCCAC
sg*GPX4*#1	CACCGGTCCCGGGACGCGCACTGCA	AAACTGCAGTGCGCGTCCCGGGACC
sg*TM7SF2*#1	CACCGGGGCCCTCCGAATTCCAGCG	AAACCGCTGGAATTCGGAGGGCCCC
sg*TM7SF2*#2	CACCGCTACCTACTGCCGGCGCGCA	AAACTGCGCGCCGGCAGTAGGTAGC
sg*LBR*#1	CACCGCCAAGTAGGAAATTTGCCGA	AAACTCGGCAAATTTCCTACTTGGC
sg*LBR*#2	CACCGTGGTAAGAGGTCGATGGCCT	AAACAGGCCATCGACCTCTTACCAC
sg*EBP2*#1	CACCGGGGTCTTAGTCGTGACCACA	AAACTGTGGTCACGACTAAGACCCC
sg*EBP2*#2	CACCGGTGCTGCGGTTGTCCCATTG	AAACCAATGGGACAACCGCAGCACC
sg*SC5D*#1	CACCGTCAGAAGACTAATAGCTTGT	AAACACAAGCTATTAGTCTTCTGAC
DHCR7Left1	CACCGCTGCATCCGTCCTCGCAGGG	AAACCCCTGCGAGGACGGATGCAGC
DHCR7Left2	CACCGCTCAGAATATGAGCGGAGGT	AAACACCTCCGCTCATATTCTGAGC
DHCR7Left3	CACCGCATTTCGCCATAGAACCATG	AAACCATGGTTCTATGGCGAAATGC
DHCR7Right1	CACCGGTATCAAACGCTGATGTGAC	AAACGTATCAAACGCTGATGTGACC
DHCR7Right2	CACCGGGATAGGAGCAAAACCCCGT	AAACACGGGGTTTTGCTCCTATCCC
DHCR7Right3	CACCGCGTGACTCCTGCTGACAATG	AAACCATTGTCAGCAGGAGTCACGC
mDhcr7Left	CACCGTGTGTCTGTCCACTAAAACC	AAACGGTTTTAGTGGACAGACACAC
mDhcr7Right	CACCGGCATCCAAAAGGCTGATCCC	AAACGGGATCAGCCTTTTGGATGCC

Supplementary Table 2. Primers used for real-time RT-PCR analysis

	Forward	Reverse
ACSL4	CCCTGAAGGATTTGATCTCTTCG	CCTTAGGTCGGCCAGTAGAAC
AIFM2	GGCCAACATCGTCAACTCTG	ACACCGTCATTTCTCCCCAT
DHCR7	ATGCCGTCTCCACCTTCG	AACCACTTCCCGATCCGA
DHODH	CCACGGGAGATGAGCGTTTC	CAGGGAGGTGAAGCGAACA
FDPS	AGGAATTGATGGCGAGAAGG	CCCAAAGAGGTCAAGGTAATCA
GPX4	GCCTTCCCGTGTAACCAGT	GCGAACTCTTTGATCTCTTCG
HMGCR	GTTCTGAACTGGAACATGGGC	TTCATCCTCCACAAGACAATGC
HMGCS1	CATTAGACCGCTGCTATTCTGTC	TTCAGCAACATCCGAGCTAGA
LDLR	AGTTGGCTGCGTTAATGTGAC	TGATGGGTTCATCTGACCAGT
PCYT1A	GTGCGGGATTATGATGTGTATG	CCTCTCCTGCAAGTGGTATTTC
PCYT2	GAGAGGCCCTACATCATCGC	ACGCTCAGAGTCCGTTCATG
PEMT	TGGATCCCAGCTTTGTGG	GCTTGCGGGTCTTGTGTT
SC5D	CTTCTGTGCAACACTGAGCTA	TCTCTCGACGGACTTGATTCTTT
SLC7A11	ATGCAGTGGCAGTGACCTTT	GGCAACAAAGATCGGACTG
SREBF2	TGTGTATGTCCTGTGCCTTTTC	TGGGACACAGTGACTGATTGAT
ACTB	GGCACTCTTCCAGCCTTCCTTC	GCGGATGTCCACGTCACACTTCA
mDhcr7	CGTCCAAGAAGGTGCCATTA	GCGTTCACAAACCAGAGGAT
mDhcr7 (Truncated)	GGTCAGCTTCCAGCAAATTC	AGTCAAACCACTTCCCGATG
mCyp2e1	GGGACATTCCTGTGTTCCAG	CTTAGGGAAAACCTCCGCAC
mActb	CACAGCTTCTTTGCAGCTCCTT	AGCGCAGCGATATCGTCAT

Supplementary Table 3. Primers used in first PCR for amplicon sequencing

	Sequence
DHCR7#1-forward	TCGTCGGCAGCGTCAGATGTGTATAAGAGACAGTTTTAAGCAAATTCACAGGCAAT
DHCR7#1-reverse	GTCTCGTGGGCTCGGAGATGTGTATAAGAGACAGGCCCATTGAAGAACAGCTTG
DHCR7#2-forward	TCGTCGGCAGCGTCAGATGTGTATAAGAGACAGGGACATCGTCACCGGACAT
DHCR7#2-reverse	GTCTCGTGGGCTCGGAGATGTGTATAAGAGACAGGGAAGGTGACCCACAAGGTA
SC5D-forward	TCGTCGGCAGCGTCAGATGTGTATAAGAGACAGTGACTGTTCTTAAATCAGGAACTTG
SC5D-reverse	GTCTCGTGGGCTCGGAGATGTGTATAAGAGACAGTTAATGCATGATCGAAGACAAAA
TM7SF2#1 - forward	TCGTCGGCAGCGTCAGATGTGTATAAGAGACAGGCGGACAGTGTTTCCTTGAC
TM7SF2#1 - reverse	GTCTCGTGGGCTCGGAGATGTGTATAAGAGACAGTCAGCTCTCCCTCTCCAGAC
TM7SF2#2 - forward	TCGTCGGCAGCGTCAGATGTGTATAAGAGACAGGCTGGAGGTGCTGTGGAG
TM7SF2#2 - reverse	GTCTCGTGGGCTCGGAGATGTGTATAAGAGACAGAGGCGACTCTTGTCCTTCAA
LBR-forward	TCGTCGGCAGCGTCAGATGTGTATAAGAGACAGAGCCTGTGGAAAAAGACGAA
LBR-reverse	GTCTCGTGGGCTCGGAGATGTGTATAAGAGACAGGCTGTCGTGGCTCAGAATTT
EBP-forward	TCGTCGGCAGCGTCAGATGTGTATAAGAGACAGCCTATACACACGCAGCCATC
EBP-reverse	GTCTCGTGGGCTCGGAGATGTGTATAAGAGACAGAAATCCCATCCCACAGCATA

Supplementary Table 4. Multiple reaction monitoring transition for oxidized phospholipids

Lipid	Precursor Ion	Product Ion	Collision Energy	Polarity
PC 16:0_18:2;O2	790.55	184.05	−32	Positive
PC 16:0_20:4;OOH	814.55	184.05	−32	Positive
PC 16:0_22:6;OOH	838.55	184.05	−32	Positive
PC 18:0_20:4;O	826.6	184.05	−32	Positive
PC 18:0_20:4;O3	858.6	184.05	−32	Positive
PC 16:0_9:0;oxo	650.45	184.05	−35	Positive
PC 18:0_9:0;oxo	678.45	184.05	−35	Positive
PC 16:0_5:0;oxo	594.4	184.05	−35	Positive
PC 16:0_4:0;COOH	610.4	184.05	−35	Positive
PC 16:0_8:0;COOH	666.45	184.05	−35	Positive
PE 16:0_18:2;O	732.5	591.5	−24	Positive
PE 16:0_20:3;O2	774.55	633.55	−24	Positive
PE 16:0_22:4;O	784.6	643.6	−24	Positive
PE 16:0_22:5;O2	798.55	657.55	−24	Positive
PE 16:0_22:6;O	780.5	639.5	−24	Positive
PE 16:0_22:6;O2	796.5	655.5	−24	Positive
PE 18:0_18:2;O	760.55	619.55	−24	Positive
PE 18:0_18:2;O,OH	776.55	635.55	−24	Positive
PE 18:0_18:3;O,OH	774.5	633.5	−24	Positive
PE 18:0_20:4;O	784.55	643.55	−24	Positive
PE 18:0_20:4;2OH,O	814.5	673.5	−24	Positive
PE 18:0_20:4;O3	816.55	675.5	−24	Positive
PE 18:0_22:5;O	810.6	669.6	−24	Positive
PE 18:1_18:2;O	758.5	617.5	−24	Positive
PE 18:1_18:2;O2	774.5	633.5	−24	Positive
PE 18:1_18:3;O,OH	772.45	631.45	−24	Positive
PE 18:1_22:4;O	834.5	693.5	−24	Positive
PE 18:1_22:6;O	806.5	665.5	−24	Positive

Supplementary methods

Pfa1 experiments

4-hydroxytamoxifen-induced *Gpx4*-knockout mouse embryonic fibroblasts (MEFs; Pfa1 cells) were maintained in DMEM-high glucose with 10% FBS. sgRNA for murine *Dhcr7* (Forward, CACCGATAGATGATGTAAAAGTAG; Reverse AAACCTACTTTTACATCATCTATC) was cloned into BsmBI-digested lentiCRISPRv2-puro vector (Addgene, #98290), and transformed into NEB stable competent cells (NEB, #C3040H). Plasmids were isolated using NcleoSpin Plasmidm Mini kit for plasmid DNA (MACHEEY-NAGEL). To produce the lentiviral particles, pHCMV-EcoEnv (addgene, #15802), psPAX2 (addgene, #12260), and vector plasmids were co-lipofected into HEK293T cells using PEI MAX (Polysciences, #24765). Viral particle-containing cell culture supernatant was collected 24 h after transfection, and Pfa1 cells were incubated in medium with 10 µg ml–1 protamine sulfate and lentivirus overnight. The cell culture medium was replaced with fresh medium containing puromycin (Gibco, #A11138-03; 1 µg ml–1), followed by selection of single-cell clones and sequencing to confirm knockout.

Human DHCR7 cDNA (NM_001360.3) was cloned from serum starved Huh-7 cells, and subcloned into p442-IRES-Blast vector. For generating SLOS-related mutants (R228Q, T93M), desired DNAs were amplified by KOD one (Sigma, #KMM-201NV), and PCR products were purified by NucleoSpin Gel and PCR Clean-up, Mini kit for gel extraction and PCR clean up (MACHEEY-NAGEL). Ligation reactions of PCR products with digested vectors were performed using in Fusion cloning enzymes (Takara Bio, #638949) according to manufacture's recommendation, Plasmids were isolated and induced in *Dhcr7*-KO Pfa1 cells using lentivirus described above. The cells were selected by blasticidin (Invitrogen, # A1113903; 10 µg ml–1), followed by experiments.

Cells were seeded on 96 well plates (2,000 cells/well) and cultured overnight. On the next day, the medium was changed to a medium containing RSL-3 with or without Lip-1 at the indicated concentrations for 24 h. Cell viability was determined by 0.004 % resazurin as an indicator of viable cells, As readout, fluorescence was measured at Ex/Em= 540/590 nm using a SpectraMax M5 microplate reader after 3.5 h of incubation, and relative viability (%) normalized to control cells were calculated.

Western blot analysis

Protein samples were separated by sodium dodecyl sulfate-polyacrylamide electrophoresis (SDS-PAGE) and transferred to PVDF membranes. After blocking with Blocking One (NACALAI

TESQUE, Kyoto, Japan) for 30 min, the membranes were incubated overnight at 4 °C with the following primary antibodies for ACSL4 (ab155282; Abcam, Cambridge, UK), AIFM2/FSP1(#24972 , Cell Signaling Technology, Danvers, MA), DHCR7 (PA5-48204; Thermo Fisher Scientific; Waltham, MA, USA), EBP (sc-374267; Santa Cruz Biotechnology, Dallas, TX, USA), DMT1(20507-1-AP; Proteintech, Rosemont, IL, USA), FTH1 (#3998; Cell Signaling Technology), FTL (10727-1-AP; Proteintech), GPX4 (ab125066; Abcam), LBR (ab32535; Abcam), SLC7A11/xCT (26864-1-AP; Proteintech), SLC40A1/Ferroportin (NBP1-21502; NOVUS, Centennial, CO), TM7SF2 (12033-1AP; Proteintech), β-actin (A5441, Sigma; St Louis, MO, USA), and VCP (ab109240, Abcam). HRP-Goat anti mouse Superclonal IgG (Thermo Fisher Scientific; Waltham, MA, USA) or HRP-goat antirabbit IgG (Cell Signaling Technology) were used as secondary antibodies and incubated with membrane for 1 h. After washing with TBS-Tween, immunoreactive bands were visualized by Western Blot Quant HRP substrate (TAKARA Bio, Shiga, Japan) or Western BLoT Ultra Sensitive HRP substrate (TAKARA Bio).

Real-time reverse transcription-polymerase chain reaction (RT-PCR)

Total RNA was prepared using ISOGEN (Nippon Gene Co., Ltd., Toyama, Japan) according to the manufacturer's instructions. Real-time RT-PCR analysis was performed using the Thermal Cycler Dice Real-Time System II (Takara Bio Inc., Shiga, Japan). The primers are listed in Supplementary Table 2. The expression levels of each target gene were normalized by subtracting the corresponding β-actin threshold cycle (CT) value; normalization was carried out using the ΔΔCT comparative method.

Heteroduplex mobility assay

The mutations in *DHCR7*-ablated cells were assessed by heteroduplex mobility assay (HMA). The genomic DNA was extracted from the cell pellet with phenol-chloroform. The mutated regions of DHCR7 were amplified with the following primers: DHCR7#1 forward, 5′-TTTTAAGCAAATTCACAGGCAAT-3′; DHCR7#1 reverse, 5′- GCCCATTGAAGAACAGCTTG-3′; DHCR7#2 forward, 5′-TTTTAAGCAAATTCACAGGCAAT-3′; DHCR7#2 reverse, 5′-GGAAGGTGACCCACAAGGTA-3′. The amplicon was denatured at 95ºC for 5 min and then

gradually cooled in a stepwise manner. The heteroduplex was separated by polyacrylamide gel electrophoresis.

LC-MS/MS analysis for 7-DHC

7-DHC analysis of culture cells: Aliquots of frozen cells were homogenized in PBS. After the lipids were extracted by methanol and chloroform, the chloroform layer was collected and evaporated at 80°C under nitrogen gas. The residue was dissolved in 100 μL of EtOH. 7-dehydroxycholesterol contents was quantified by LC-MS/MS. Briefly, [$^2H_6$]7-dehydroxycholesterol (5 ng) was added to 2μL of lipids extract as an internal standard, and saponification was carried out in 0.5 mL of 1N ethanolic KOH with butylated hydroxytoluene at 37 °C for 1 h. After the addition of 0.25 mL of distilled water, sterols were extracted with 1 mL of n-hexene, and the extract was evaporated to dryness under a nitrogen gas stream. The reagent mixture for derivatization consisted of 2-methyl-6-nitrobenzoic anhydride (100 mg), 4-dimethylamino- pyridine (30 mg), picolinic acid (80 mg), pyridine (1.5 mL), and triethylamine (200 μL). The freshly prepared reagent mixture (170 μL) was added to the sterol extract, and the reaction mixture was incubated at 80°C for 60 min. After the addition of 1 ml of n-hexane, the mixture was centrifuged at 1,500 g for 5 min. The clear supernatant was collected and evaporated at 80°C under nitrogen gas. The residue was dissolved in 50 μL of acetonitrile, and an aliquot (5 μL) was injected into the following LC-MS/MS system. The LC-MS/MS system consisted of a TSQ Quantum Ultra mass spectrometer (Thermo Fisher Scientific, San Jose, CA) equipped with an H-ESI probe and an Ultimate 3000 HPLC system (Thermo Fisher Scientific, San Jose, CA). Chromatographic separation was performed using a Hypersil GOLD column (150 3 2.1 mm, 3 mm, Thermo Electron) at 40°C, and the following gradient system was used at a flow rate of 300 ml/min: initially, the mobile phase was composed of acetonitrile-methanol-water (40:40:20, v/v/v) containing 0.1% acetic acid; then it was programmed in a linear manner to acetonitrile-methanol-water (45:45:10, v/v/v) containing 0.1% acetic acid over 20 min. The final mobile phase was kept constant for an additional 20 min. The general LC-MS/MS conditions were as follows: spray voltage, 1,000 V; vaporizer temperature, 350°C; sheath gas (nitrogen) pressure, 85 psi; auxiliary gas (nitrogen) flow, 60 arbitrary units; ion transfer capillary temperature, 350°C; collision gas (argon) pressure, 1.5 mTorr; and ion polarity, positive. The multiple ion detector was focused on m/z 553.339 for 7-

dehydroxycholesterol, and m/z 560.377 for [$^{2}H_{6}$]7-dehydroxycholesterol was used as an internal standard for 7-dehydroxycholesterol.

LC-MS/MS analysis for 7-DHC and DHCEO

7-DHC analysis of tissue samples and DHCEO analysis: The LC-MS/MS system consisted of a 4000 QTRAP tandem mass spectrometer (SCIEX; Tokyo, Japan) equipped with an Exion LC system (SCIEX). Chromatographic separation was performed using an ODS column (5C18-MS-II 5μm, 4.6×250 mm; Nacalai Tesque; Kyoto, Japan) at 40°C. The column was eluted with a mobile phase consisting of solvent A (methanol-water (90:10, v/v) containing 0.1% formic acid) and solvent B (2-propanol containing 0.1% formic acid). The mobile phase gradient profile was as follows: 0–10 min, 0% B; 10–20 min, 0–30% B linear; 20.1 min, 100% B. The flow rate was 1.0 mL/min. The general LC-MS/MS conditions were as follows: declustering potential, 71.0 V; entrance potential, 10.0 V; collision energy, 21.0 V; collision cell exit potential, 18.0 V; temperature, 500°C; source, APCI; and ion polarity, positive. DHCEO was detected by multiple reaction monitoring (MRM) for the transition of precursor ions to products: (*m/z* 399>381). Standard DHCEO (EVU139) was purchased from Kerafast (Boston, MA)

Enzymatic activities of DHCR7

Enzymatic activities of DHCR7 were determined by the conversion of ergosterol to brassicasterol. DHCR7-ablated Huh-7 Cells were treated with 2 μg/mL ergosterol for 24 h and cellular lipids were extracted by Bligh and Dyer method. Butylated hydroxytoluene and stigmasterol (5 ng, internal standard) were added before the extraction. After saponification, sterols were extracted by n-hexane as described above and dissolved in 50 μL of acetonitrile, and an aliquot (5 μL) was injected into the LC-MS/MS system (Ultimate 3000 HPLC system hyphenated with TSQ Quantum Ultra mass spectrometer equipped with APCI probe). Chromatographic separation was performed using a Hypersil GOLD column at 40°C. The column was eluted with a mobile phase consisting of solvent A (water containing 0.1% acetic acid) and solvent B (acetonitrile-methanol (50:50, v/v) containing 0.1% acetic acid). The mobile phase gradient profile was as follows: 0–20 min, 85–95% B linear; 20.1 min,

95% B; 30.1 min 85% B. The flow rate was 0.3 mL/min. The general LC-MS/MS conditions were as follows: discharge current, 4,0 μA; vaporizer temperature, 300°C; sheath gas (nitrogen) pressure, 30 psi; auxiliary gas (nitrogen) flow, 5 arbitrary units; ion transfer capillary temperature, 300°C; and ion polarity, positive. The MRM transitions of precursor ions to products are as follows: Brassicasterol (*m/z* 381>147), Ergosterol (*m/z* 379>145), Stigmasterol (*m/z* 395>147).

LC-MS/MS analysis for sterol intermediates of cholesterol synthesis

Standard zymostenol, lathosterol, 14-dehydro zymostenol were purchased from Cayman. The saponified lipid extracts were injected into the LC-MS/MS system (Ultimate 3000 HPLC system hyphenated with TSQ Quantum Ultra mass spectrometer equipped with APCI probe). Chromatographic separation was performed using a Hypersil GOLD column at 40°C. The column was eluted with acetonitrile-methanol-water (40.5:40.5:19) containing 0.1% acetic acid. The flow rate was 0.4 mL/min. The general LC-MS/MS conditions were as follows: discharge current, 4,0 μA; vaporizer temperature, 300°C; sheath gas (nitrogen) pressure, 30 psi; auxiliary gas (nitrogen) flow, 5 arbitrary units; ion transfer capillary temperature, 300°C; and ion polarity, positive. The MRM transitions of precursor ions to products are as follows: cholesterol, lathosterol, and zymostenol (*m/z* 369>95), 7-DHC (*m/z*367 >145), 14-dehydro zymostenol (*m/z* 367>254).

Thin-layer chromatography

Thin-layer chromatography (TLC) was used to separate the total lipids extract obtained by the Bligh and Dyer method, which was resolved in chloroform/methanol (2:1, v/v). The TLC plate (1.05553, Sigma) were developed using either n-heptane/etylacetate (2:1,v/v) or chloroform/methanol/water (65:25:4) as the solvent system. After development, the plates were immersed in a solution containing 60 mL of methanol, 60 mL of water, 0.4 g of MnCl2 and 4 mL of sulfuric acid and then heated in an oven at 105ºC for 10 min. The spots were visualized and quantified using Amersham ImageQuant800 (Cytiva).
