# Supplementary material for: Inhibition of 7-dehydrocholesterol reductase prevents hepatic ferroptosis under an active state of sterol synthesis
Source: Nat Commun. 2024 Mar 12;15:2195. doi: 10.1038/s41467-024-46386-6 (PMC10933264; doi:10.1038/s41467-024-46386-6)
Supplement: Supplementary file 3 — Reporting Summary [file 41467_2024_46386_MOESM3_ESM.pdf]

nature portfolio

Corresponding author(s): Naoya Yamada
Tadayoshi Karasawa
Masafuimi Takahashi

Last updated by author(s): Feb 7, 2024

Reporting Summary

Nature Portfolio wishes to improve the reproducibility of the work that we publish. This form provides structure for consistency and transparency in reporting. For further information on Nature Portfolio policies, see our Editorial Policies and the Editorial Policy Checklist.

Statistics

For all statistical analyses, confirm that the following items are present in the figure legend, table legend, main text, or Methods section.

n/a	Confirmed	
☐	☒	The exact sample size (*n*) for each experimental group/condition, given as a discrete number and unit of measurement
☐	☒	A statement on whether measurements were taken from distinct samples or whether the same sample was measured repeatedly
☐	☒	The statistical test(s) used AND whether they are one- or two-sided<br>*Only common tests should be described solely by name; describe more complex techniques in the Methods section.*
☒	☐	A description of all covariates tested
☐	☒	A description of any assumptions or corrections, such as tests of normality and adjustment for multiple comparisons
☐	☒	A full description of the statistical parameters including central tendency (e.g. means) or other basic estimates (e.g. regression coefficient) AND variation (e.g. standard deviation) or associated estimates of uncertainty (e.g. confidence intervals)
☐	☒	For null hypothesis testing, the test statistic (e.g. *F*, *t*, *r*) with confidence intervals, effect sizes, degrees of freedom and *P* value noted<br>*Give P values as exact values whenever suitable.*
☒	☐	For Bayesian analysis, information on the choice of priors and Markov chain Monte Carlo settings
☒	☐	For hierarchical and complex designs, identification of the appropriate level for tests and full reporting of outcomes
☒	☐	Estimates of effect sizes (e.g. Cohen's *d*, Pearson's *r*), indicating how they were calculated

Our web collection on statistics for biologists contains articles on many of the points above.

Software and code

Policy information about availability of computer code

Data collection	FV10-ASW ver.03.00 (Olympus), FACSuite(BD Biosciences), Spark Control v2.3 (TECAN), i-control 2.0 (TECAN), Image Quant LAS 4000 mini version 1.0 (GE healthcare), Amersham Imager 680 ver 2.0.0 (Cytiva), Amersham Image Quant800 ver 1.2.0 (Cytiva), Thermal Cycler Dice Real Time System Software ver 5.11B (TAKARA), Thermo Xcalibur 2.2 (Thermo Fisher Scientific), LabSolutions version 5.80 (Shimazu), 4000 QTRAP (Sciex).
Data analysis	GraphPad Prism v7 (GraphPad Software), R version 4.1.1 (https://www.r-project.org), MAGeCK(0.5.4), HISAT2-2.2.1, DEseq2 R packages (version 1.32.0), featureCounts (subread 2.0.1), GSEA4.1.0 (http://www.gsea-msigdb.org/gsea/index.jsp), FlowJo software (version 10), FIJI/ Image J (2.1.0/1.53c), Analyst 1.7.2 (Sciex).

For manuscripts utilizing custom algorithms or software that are central to the research but not yet described in published literature, software must be made available to editors and reviewers. We strongly encourage code deposition in a community repository (e.g. GitHub). See the Nature Portfolio guidelines for submitting code & software for further information.

Data

Policy information about availability of data

All manuscripts must include a data availability statement. This statement should provide the following information, where applicable:

- Accession codes, unique identifiers, or web links for publicly available datasets
- A description of any restrictions on data availability
- For clinical datasets or third party data, please ensure that the statement adheres to our policy

All data are available in the Article and the Supplementary Information, and from the corresponding author on reasonable request. The sequence data generated in this study have been deposited in the Gene Expression Omnibus dataset (accession number: GSE228883).Human cancer cell line data were obtained from CCLE (https://sites.broadinstitute.org/ccle/) .Human protein and gene expression were obtained from RefEx (https://refex.dbcls.jp/)

Research involving human participants, their data, or biological material

Policy information about studies with human participants or human data. See also policy information about sex, gender (identity/presentation), and sexual orientation and race, ethnicity and racism.

Reporting on sex and gender	n.a.
Reporting on race, ethnicity, or other socially relevant groupings	n.a.
Population characteristics	n.a.
Recruitment	n.a.
Ethics oversight	n.a.

Note that full information on the approval of the study protocol must also be provided in the manuscript.

Field-specific reporting

Please select the one below that is the best fit for your research. If you are not sure, read the appropriate sections before making your selection.

☒ Life sciences ☐ Behavioural & social sciences ☐ Ecological, evolutionary & environmental sciences

For a reference copy of the document with all sections, see nature.com/documents/nr-reporting-summary-flat.pdf

Life sciences study design

All studies must disclose on these points even when the disclosure is negative.

Sample size	Sample sizes in vitro experiments were determined based on the numbers required to achieve statistical significance using indicated statistics, as well as considering of previous publications on similar experiments (PMID: 31634899 and 35922516).
Data exclusions	No data exclusions.
Replication	The experimental findings were reproduced in multiple independent experiments. The number of biological replicates and independent experiments are presented in each figure legends.
Randomization	For animal experiments, mice with same age were randomly grouped into experiments. Randomization is not relevant to the in vitro experiments since cells come in millions of populations and are automatically randomized and seeded to different wells for treatment.
Blinding	In the in vitro experiments, the investigators were not blinded, which is standard in this type of study due to the multiple steps involved that require precise operations for accuracy and precision precluding blinding to experimental variables.

Reporting for specific materials, systems and methods

We require information from authors about some types of materials, experimental systems and methods used in many studies. Here, indicate whether each material, system or method listed is relevant to your study. If you are not sure if a list item applies to your research, read the appropriate section before selecting a response.

Materials & experimental systems

n/a	Involved in the study	
☐	☒	Antibodies
☐	☒	Eukaryotic cell lines
☒	☐	Palaeontology and archaeology
☐	☒	Animals and other organisms
☒	☐	Clinical data
☒	☐	Dual use research of concern
☒	☐	Plants

Methods

n/a	Involved in the study	
☒	☐	ChIP-seq
☐	☒	Flow cytometry
☒	☐	MRI-based neuroimaging

Antibodies

Antibodies used

ACSL4 (ab155282; Abcam, Cambridge, UK), AIFM2/FSP1(#24972 , Cell Signaling Technology, Danvers, MA), DHCR7 (PA5-48204; Thermo Fisher Scientific; Waltham, MA, USA), EBP (sc-374267; Santa Cruz Biotechnology, Dallas, TX, USA), DMT1(20507-1-AP; Proteintech, Rosemont, IL, USA), FTH1 (#3998; Cell Signaling Technology), FTL (10727-1-AP; Proteintech), GPX4 (ab125066; Abcam), LBR (ab32535; Abcam), SLC7A11/xCT (26864-1-AP; Proteintech), SLC40A1/Ferroportin (NBP1-21502; NOVUS, Centennial, CO), β-actin (A5441, Sigma; St Louis, MO, USA), VCP (ab109240, Abcam), HRP-Goat anti mouse Superclonal IgG (Thermo Fisher Scientific; Waltham, MA, USA), HRP-goat antirabbit IgG (Cell Signaling Technology)

Validation

All primary antibodies were used for human or mouse W.B. in previous articles.
ACL4 (PMID: 30962421), AIFM2/FSP1 (PMID: 36788244), DHCR7 (PMID: 33284321), DMT1(PMID: 33529321), EBP(PMID: 30535733), FTH1(PMID: 34155415),FTL(0727-1-AP), GPX4 (PMID: 31634899), LBR (PMID: 33846636), SLC7A11/xCT (PMID: 36864172),SLC40A1/Ferroportin (PMID: 31585094)

Eukaryotic cell lines

Policy information about cell lines and Sex and Gender in Research

Cell line source(s)

Huh-7 (human hepatocellular carcinoma), OVISE (human ovarian clear cell carcinoma), and HT-1080 (human fibrosarcoma) cells were obtained from the Japanese Collection of Research Biosources (JCRB) Cell Bank (Japan). PLC/PRF/5 and HLE (human hepatocellular carcinoma) cells were obtained from The Health Science Research Resource Bank (HSRRB, Japan). SKHep1 (human hepatocellular carcinoma) cells were obtained from ATCC. Pfa1 (4-hydroxytamoxifen-induced Gpx4-knockout mouse embryonic fibroblasts) cells were kindly provided by Marcus Conrad.

Authentication

None of the cell lines used were authenticated.

Mycoplasma contamination

All cell lines were tested negative for mycoplasma contamination.

Commonly misidentified lines
(See ICLAC register)

No commonly misidentified cell lines were used.

Animals and other research organisms

Policy information about studies involving animals; ARRIVE guidelines recommended for reporting animal research, and Sex and Gender in Research

Laboratory animals

Colonized specific pathogen free (SPF) mice on a C57BL/l background were purchased from SLC Japan (Shizuoka, Japan). Mice were housed (4/cage, RAIR HD ventilated Micro-Isolator Animal Housing Systems, Lab Products, Seaford, DE) in an environment maintained at 23 ± 2C with ad libitum access to food and water under a 12-h light/dark cycle with lights on from 8:00 to 20:00.

Wild animals

No wild animal were used in this study.

Reporting on sex

All mice were male. No conclusions with respect to sex difference can be made.

Field-collected samples

No field-collected samples were used in this study.

Ethics oversight

All experiments in this study were performed in accordance with the Jichi Medical University Guide for Laboratory Animals (Permit Nos. 17141-02 and 20107-02).

Note that full information on the approval of the study protocol must also be provided in the manuscript.

Flow Cytometry

Plots

Confirm that:

☒ The axis labels state the marker and fluorochrome used (e.g. CD4-FITC).

☒ The axis scales are clearly visible. Include numbers along axes only for bottom left plot of group (a 'group' is an analysis of identical markers).

☒ All plots are contour plots with outliers or pseudocolor plots.

☒ A numerical value for number of cells or percentage (with statistics) is provided.

Methodology

Sample preparation	Assessment of lipid peroxidation using C11-BODIPY581/591. Huh-7 cells (100,000 cells/well) were seeded on 12 well plates. Cells were cultured overnight and labeled with 1 µM C11-BODIPY581/591 (#D3861; Thermo Fisher Scientific) for 1 h before RSL-3 (0.1 µM) treatment. After the treatment, cells were detached with TrypLE Express (Gibco) and resuspended with PBS containing 2%FCS. Cells strained through a 35 µm cell strainer (352235, FALCON) were analyzed by flow cytometry (FACS Verse, BD Biosciences).
Instrument	FACS Verse, BD Biosciences
Software	For data collection, BD FACSuite software was used. For data analysis FlowJo (Version 10) was used.
Cell population abundance	A total of 4,000-10,000 cells were analyzed for each sample.
Gating strategy	Cellular debris and doublets were excluded using FSC and SSC .

☐ Tick this box to confirm that a figure exemplifying the gating strategy is provided in the Supplementary Information.
